# Gingerol-Enriched Ginger Extract Effects on Anxiety-like Behavior in a Neuropathic Pain Model via Colonic Microbiome-Neuroimmune Modulation

**DOI:** 10.3390/molecules31010166

**Published:** 2026-01-01

**Authors:** Roberto Mendóza, Julianna M. Santos, Xiaobo Liu, Moamen M. Elmassry, Guangchen Ji, Takaki Kiritoshi, Volker Neugebauer, Chwan-Li Shen

**Affiliations:** 1 Department of Pathology, Texas Tech University Health Sciences Center, Lubbock, TX 79430, USA; robert.mendoza@ttuhsc.edu (R.M.); julianna.santos@ttuhsc.edu (J.M.S.); xiaobo.liu@ttuhsc.edu (X.L.); 2 Department of Internal Medicine, Texas Tech University Health Sciences Center, Lubbock, TX 79430, USA; 3 Woody L. Hunt School of Dental Medicine, Texas Tech University Health Sciences Center, El Paso, TX 79905, USA; 4 Department of Molecular Biology, Princeton University, Princeton, NJ 08540, USA; elmassry@princeton.edu; 5 Department of Pharmacology and Neuroscience, Texas Tech University Health Sciences Center, Lubbock, TX 79430, USA; guangchen.ji@ttuhsc.edu (G.J.); takaki.kiritoshi@ttuhsc.edu (T.K.); volker.neugebauer@ttuhsc.edu (V.N.); 6 Center of Excellence for Translational Neuroscience and Therapeutics, Texas Tech University Health Sciences Center, Lubbock, TX 79430, USA; 7 Garrison Institute on Aging, Texas Tech University Health Sciences Center, Lubbock, TX 79430, USA; 8 Center of Excellence for Integrative Health, Texas Tech University Health Sciences Center, Lubbock, TX 79430, USA

**Keywords:** bioactive compounds, anxiety, brain, pain, rats, gut–brain axis, colon, microbiome, neuroimmune, neuropathic pain, colon

## Abstract

Growing evidence has revealed that gut dysbiosis is associated with the development of anxio-depressive disorders through mechanisms that involve neuroimmune signaling, neurotransmitter changes, and neuroplasticity in the brain. This study investigated the effects of gingerol-enriched ginger (GEG) on specifically anxiety-related neuroinflammation-, neuroimmunity-, neuroplasticity-, neurotransmission-, and neurotoxicity-associated genes in different brain regions, as well as on alterations linked to colonic microflora-driven dysbiosis, in the spinal nerve ligation (SNL) rat model of neuropathic pain (NP). Twenty-seven male rats were assigned to 3 groups: sham, SNL, and SNL-treated with GEG at 200 mg/kg body weight (SNL+200GEG) via oral gavage for 5 weeks. Anxiety-like behavior was assessed on the elevated plus maze (EPM). mRNA expression was assessed by qRT-PCR using respective primers. Correlation between behavioral parameters and colonic microbiome composition was analyzed using the Spearman rank correlation. The SNL+200GEG group demonstrated decreased anxiety-like behavior in the SNL model. Compared to the SNL group, the SNL+200GEG group had increased mRNA expression of NRF2 (amygdala: left), LXRα (amygdala: both sides), and CX3CR1 (amygdala: both sides, hippocampus: right). GEG modulated neuroplasticity as shown by increased gene expression of PGK1 (amygdala: right, hippocampus: both sides), MEK1 (frontal cortex: both sides), LDHA (frontal cortex: both sides), GPM6A (frontal cortex: both sides, amygdala: right, hippocampus: right, and hypothalamus), and GLUT1 (amygdala: right) as well by decreased gene expression of HIF1α (in all brain regions except for the hypothalamus). GEG modulated neurotransmission via clearance of excessive glutamate release as suggested by increased gene expression of SLC1A3 (frontal cortex: both sides, hippocampus: right) and via augmenting mGluR5 signaling as shown by increased gene expression of GRM5 (hippocampus: both sides, hypothalamus) as well as downregulation of KMO, HAAO, GRIN2B, and GRIN2C influencing downstream serotonergic neurotransmission and NMDA receptor-mediated glutamatergic pathways in different brain regions. GEG further alleviated neurotoxicity through downregulated gene expression of SIRT1, KMO, IDO1, and HAAO in different brain regions. Moreover, the increased relative abundance of *Bilophila* spp., accompanied by decreased time spent in the EPM open arms, suggests that increased *Bilophila* abundance increases anxiety-like behavior. GEG supplementation mitigated anxiety-like behavior in male rats with NP, at least in part, by reducing SNL-induced inflammatory sequelae-related mRNA gene expression in different brain regions. In addition, there is a positive correlation between the abundance of *Bilophila wadsworthia* and the degree of anxiety-like behavior.

## 1. Introduction

Chronic neuropathic pain is a major health concern and a complex disorder with sensory and affective dimensions, the latter often manifesting as anxiety and depression [1]. These affective disturbances are attributed, at least in part, to neuroinflammatory processes in brain regions regulating mood and stress responses. Emerging evidence also implicates gut dysbiosis in this cascade, as alterations in microbial composition and barrier function can promote systemic immune activation and drive neuroinflammation. Thus, neuropathic pain (NP) provides a clinically relevant context in which to examine how dysbiosis-related immune signaling contributes to both pain behaviors and associated anxiety-like outcomes within the inflammatory continuum. The National Comorbidity Study Replication (NCS-R) estimates that 19.1% of (approximately 1 in 5) adults in the U.S. dealt with anxiety in 2024, with higher prevalence in females (23.4%) than in males (14.3%) [2]. It is estimated that 31.1% of all adults in the U.S. experience a form of anxiety at some time in their lives [2], making anxiety both widespread and linked to substantial personal, societal, and economic costs [3,4].

Although the underlying pathophysiology of mood and anxiety disorders remains unclear, emerging studies have suggested that chronic low-grade inflammation within both the central and peripheral nervous systems (CNS and PNS, respectively), broadly referred to as neuroinflammation, may be instrumental in the onset and progression of mental health disorders, including those under the anxiety umbrella, with gut dysbiosis as the inciting factor [5,6]. This inflammatory milieu can disrupt synaptic signaling, promote CNS and PNS neurotoxicity, and compromise neuroimmune response, brain neuroplasticity, and neurotransmitter regulation [5,6]. Such evidence highlights how inflammation and oxidative stress caused by anxiety disorders disrupt neurochemical homeostasis for neurotransmitters. Oxidative stress promotes the degradation of dopaminergic neurons and altered serotonergic, glutamatergic, and dopaminergic pathways by modifying tryptophan metabolism [7,8]. Both inflammation and oxidative stress also affect the hypothalamic–pituitary–adrenal (HPA) axis, which fulfills important homeostatic functions by regulating levels of cortisol, the primary stress hormone [9]. Proinflammatory cytokines stimulate the HPA axis by inducing corticotropin-releasing and adrenocorticotropic hormones, ultimately releasing cortisol from the adrenal glands into the bloodstream. Patients with depression often exhibit chronically elevated serum cortisol levels [10]. Conversely, reduced cortisol release correlates with improved anxious and depressive clinical symptomatology [11].

Neuroimmune interactions involve microglia, the primary resident immune cells of the CNS [12,13]. Microglia function as primary effectors in responding to injury and infection, activity-dependent modulation of synaptic plasticity, and in developmental synaptic pruning [14]. In the progression of anxio-depressive states, aberrant microglial activation contributes to chronic neuroinflammation, which exacerbates neuronal dysfunction, impaired connectivity, and subsequent behavioral abnormalities [12,13]. 

Expanding upon this neuroimmune framework, the gut–brain axis has received more attention in recent years, particularly regarding the role of the gut microbiome in shaping CNS immune responses and influencing behavior [13,14]. Disruption of the microbial ecosystem, termed dysbiosis, plays a role in heightened systemic inflammation, increased intestinal permeability, and altered microbial metabolites, all of which can affect neuroinflammatory pathways [15].

Growing clinical and preclinical findings support these links. Several human studies have shown that patients with anxiety and depressive disorders frequently exhibit altered gut microbiome profiles, increased intestinal permeability, and elevated circulating inflammatory cytokines, all of which correlate with symptom severity [16,17]. Likewise, animal models in which the gut microbiome is experimentally disrupted, through antibiotics, germ-free conditions, or fecal microbiota transfer, develop heightened anxiety-like and depressive-like behaviors and increased CNS inflammatory signaling [17,18,19]. These findings suggest that microbial dysbiosis functions not only as a correlate, but as a potential driver, of emotional disturbances through peripheral immune activation and neuroinflammation. Such converging evidence highlights why neuropathic pain, which both induces or exacerbates dysbiosis and upregulates inflammatory cytokine cascades, represents a relevant model in which to investigate gut-mediated mechanisms contributing to pain-associated anxiety. Mechanistically, such immune activation can upregulate the enzyme indoleamine 2,3-dioxygenase 1 (IDO1), diverting tryptophan from serotonin synthesis toward neurotoxic kynurenine metabolites (e.g., 3-hydroxykynurenine, quinolinic acid) that impair synaptic plasticity and drive excitotoxicity, a process observed in both human and rodent models of mood disorders [18,19,20,21].

Given the connection between gut dysbiosis and anxio-depressive behavior, there is a growing interest in adjuvant pharmacological treatments or natural interventions, such as ginger, that target the gut-neuroimmune interface [22,23]. Ginger and its bioactive compounds [gingerols (6-gingerol, 8-gingerol, 10-gingerol, and 12-gingerol), shogaols (6-shogaol, 8-shogaol, 10-shogaol), gingerdiols, and paradols] possess neuroprotective effects due to their antioxidant, anti-inflammatory, and neuroprotective properties which could explain their anxiolytic and anti-depressive effects [24]. In an animal model of depression, ginger-treated rats showed a significant increase in brain serotonin availability as a result of increased serotonin synthesis and release, serotonin turnover, and decreased tryptophan 2,3-dioxygenase activity [25]. Furthermore, 6-shogaol supplementation suppresses key inflammatory mediators [e.g., tumor necrosis factor alpha (TNF-α), interleukin-1β (IL-1β)] and restores hippocampal brain-derived neurotropic factor (BDNF) in injured brain tissue, consistent with improved affective outcomes [26]. These findings underscore the capacity for ginger to modulate serotonergic tone, neurotrophic support, oxidative stress, and inflammatory cascades, providing a mechanistic rationale for its anxiolytic potential. Importantly, our prior study reported that dietary gingerol-enriched ginger (GEG) extract decreases mechanosensitivity, anxio-depressive states, and neuroimmune cell proliferation by improving gut microbiome composition and mitochondrial homeostasis in rats with diabetic neuropathy [27]. Dietary GEG supplementation attenuated anxiety-like behaviors, partly by modulating gut microbiota and fecal metabolites in animals with NP [23].

However, it is not clear how ginger and its bioactive compounds affect different brain regions in animals with anxiety-like behavior via the aforementioned molecular mechanisms. It is also unclear how ginger affects the association between the colonic microbiome and genes associated with anxio-depressive states in rats with NP. Thus, the goal of this study was to measure GEG regulation of anxiety-like behaviors and effects on pertinent molecular pathways in different brain regions of rats with NP. We hypothesized that administration of GEG to rats with NP would attenuate anxiety-like behavior by modulating the genes linked to neuroinflammation, neuroimmunity, neuroplasticity, neurotransmission, and neurotoxicity across 7 different brain regions. Furthermore, we explored the correlation between gut microbiome composition [28] and anxiety-like behaviors from previously published research to further understand how the gut–brain axis contributes to these phenomena.

## 2. Results

### 2.1. Anxiety-like Behavior

**Figure 1:**
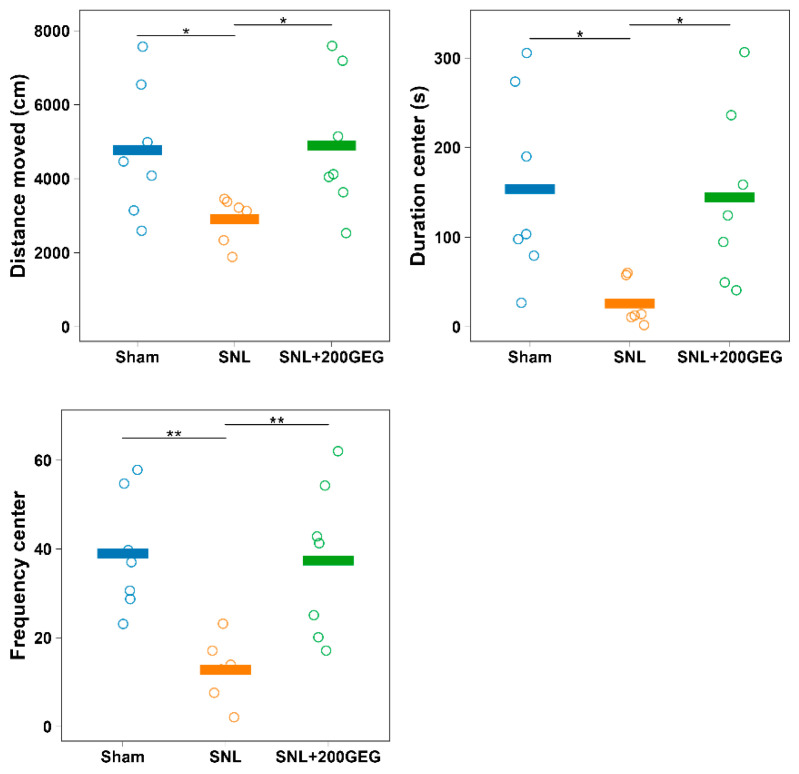
Effect of GEG supplementation on anxiety-like behavior. N = 6–8 per group. Data were analyzed by one-way ANOVA followed by the *post hoc* Tukey test at each time point. * *p* < 0.05. ** *p* < 0.01. Note: the *y*-axis label ‘center’ in the duration graph reflects a legacy label in the dataset; in the EPM paradigm, this corresponds to open-arms measures.

We utilized the elevated plus maze (EPM) test as an outcome measure for anxiety-like behavior. Compared to the sham group, the spinal nerve ligation (SNL) group traveled a shorter distance, spent less time, and performed fewer entries in the open arms (Figure 1), where the number of entries represented a measure of frequency. GEG supplementation reduced SNL-induced anxiety-like behavior of rats, as shown by increased distance, time, and frequency in the open arms (Figure 1).

### 2.2. Neuroinflammation and Neuroimmunity

**Figure 2:**
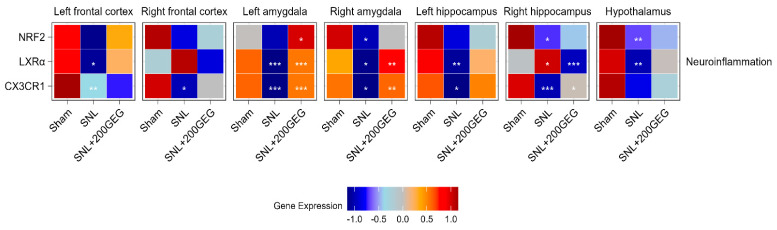
Effect of GEG supplementation on neuroinflammation mRNA expression. N = 6–8 per group. Data were analyzed by one-way ANOVA followed by the *post hoc* Tukey test at each time point for sham vs. SNL and SNL vs. SNL+200GEG for the respective gene. * *p* < 0.05. ** *p* < 0.01. *** *p* < 0.001.

Figure 2 illustrates the impact of GEG supplementation on neuroinflammatory markers, namely, the mRNA NRF2, LXRα, and CX3CR1 in different brain regions of SNL rats. Relative to the sham group, the SNL group exhibited decreased NRF2 in the amygdala (right), hippocampus (right), and hypothalamus. GEG supplementation increased NRF2 in the amygdala (left). Relative to the sham group, the SNL group exhibited decreased LXRα in the frontal cortex (left), amygdala (both sides), hippocampus (left), and hypothalamus, while LXRα was increased in the hippocampus (right). Administration of GEG increased LXRα in the amygdala (both sides) but decreased LXRα in the hippocampus (right) compared to the untreated SNL group. The untreated SNL group had decreased CX3CR1 in the frontal cortex (both sides), amygdala (both sides), and hippocampus (both sides) than the sham group. GEG treatment had increased CX3CR1 in the amygdala (both sides) and hippocampus (right) compared to the untreated SNL group.

### 2.3. Neuroplasticity

**Figure 3:**
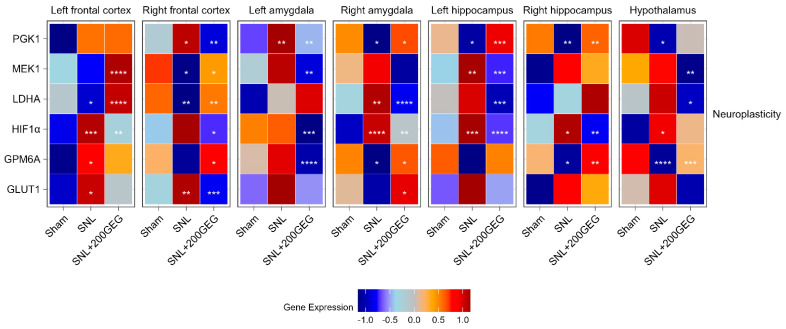
Effect of GEG supplementation on neuroplasticity genes mRNA expression. N = 6–8 per group. Data were analyzed by one-way ANOVA followed by the *post hoc* Tukey test at each time point for sham vs. SNL and SNL vs. SNL+200GEG for the respective gene. * *p* < 0.05. ** *p* < 0.01. *** *p* < 0.001. **** *p* < 0.0001.

Figure 3 illustrates the impact of GEG supplementation on neuroplasticity markers, namely, the mRNA PGK1, MEK1, LDHA, HIF1α, GPM6A, and GLUT1. Relative to the sham group, the untreated SNL group had elevated PGK1 in the frontal cortex (right) and amygdala (left), while it suppressed PGK1 in the amygdala (right), hippocampus (both sides), and hypothalamus of the rats. The GEG supplementation reverted these SNL-induced changes, except in the hypothalamus. Compared to the sham group, the SNL group had decreased MEK1 in the frontal cortex (right) and increased MEK1 in the hippocampus (left). The effects of GEG on MEK1 were brain region-specific. For example, GEG increased MEK1 in the frontal cortex (both sides) but decreased MEK1 in the amygdala (left), hippocampus (left), and hypothalamus. The SNL group had decreased LDHA in the frontal cortex (both sides) and increased LDHA in the amygdala (right) compared to the sham group. GEG supplementation increased LDHA in the frontal cortex (both sides) and decreased LDHA in the amygdala (right), hippocampus (left), and hypothalamus of the SNL rats when compared to the untreated SNL group. Relative to the sham group, the SNL group had increased HIF1α in 5 out of the 7 brain regions, the two unaffected regions being the frontal cortex (right) and amygdala (left). Administration of GEG suppressed SNL-induced HIF1α changes in the brain regions where they were measured, except in the hypothalamus. Compared to the sham group, the SNL group had increased GPM6A in the frontal cortex (left) but decreased GPM6A in the amygdala (right), hippocampus (right), and hypothalamus. GEG increased the mRNA expression of GPM6A in the frontal cortex (right), amygdala (right), hippocampus (right), and hypothalamus, but decreased in the amygdala (left) of treated SNL rats. The SNL group exhibited increased GLUT1 in the frontal cortex (both sides) compared to the sham group. Administration of GEG to SNL rats suppressed GLUT1 in the frontal cortex (right) but increased GLUT1 in the amygdala (right).

### 2.4. Neurotransmission

**Figure 4:**
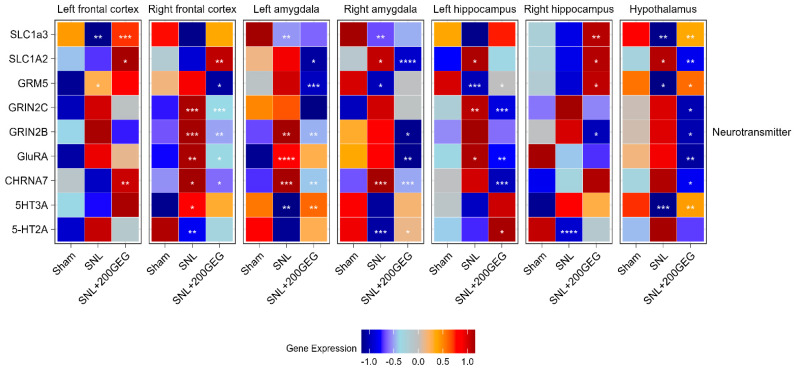
Effect of GEG supplementation on neurotransmitter mRNA expression. N = 6–8 per group. Data were analyzed by one-way ANOVA followed by the *post hoc* Tukey test at each time point for sham vs. SNL and SNL vs. SNL+200GEG for the respective gene. * *p* < 0.05. ** *p* < 0.01. *** *p* < 0.001. **** *p* < 0.0001. Note: GRIA1 labeled as GluRA in figure.

Figure 4 illustrates the impact of GEG supplementation on neurotransmission gene markers, namely, the mRNA SLC1A3 (EAAT1), SLC1A2 (EAAT2), GRM5, GRIN2B, GRIN2C, GRIA1, CHRNA7, 5-HT3A, and 5-HT2A. Relative to the sham group, the SNL group showed decreased SLC1A3 in the frontal cortex (left), amygdala (both sides), and hypothalamus. Relative to the baseline SNL group, GEG increased SLC1A3 in the frontal cortex (left), hippocampus (right), and hypothalamus. The untreated SNL group had increased SLC1A2 in the amygdala (right), hippocampus (left), and hypothalamus. Relative to the untreated SNL group, GEG decreased SLC1A2 in the amygdala (both sides) and hypothalamus (right), and increased SLC1A2 in the frontal cortex (both sides) and hippocampus (right). The SNL group had increased GRM5 in the frontal cortex (left) and decreased GRM5 in the amygdala (right), hippocampus (left), and hypothalamus compared to the sham group. Meanwhile, compared to the untreated SNL group, the GEG group suppressed GRM5 in the frontal cortex (right) and amygdala (left), but increased GRM5 in the hippocampus (both sides) and hypothalamus. Relative to the sham group, the SNL group demonstrated increased GRIN2C in the frontal cortex (right) and hippocampus (left). GEG supplementation significantly decreased GRIN2C in the frontal cortex (right), hippocampus (left), and hypothalamus of treated SNL rats. GRIN2B was increased in the frontal cortex (right) and amygdala (left) of the SNL group relative to the sham group. GEG decreased GRIN2B in the frontal cortex (right), amygdala (both sides), hippocampus (right), and hypothalamus of treated SNL rats. Relative to the sham group, the SNL group exhibited increased GRIA1 (GluRA in Figure 4) in the frontal cortex (right), amygdala (left), and hippocampus (left). GEG decreased GRIA1 in the frontal cortex (right), amygdala (right), hippocampus (left), and hypothalamus. The untreated SNL group had increased CHRNA7 in the frontal cortex (right) and amygdala (right and left) compared to the sham group. GEG increased CHRNA7 in the left frontal cortex while decreasing CHRNA7 in the right frontal cortex, amygdala (both sides), hippocampus (left), and hypothalamus. The SNL group had increased 5-HT3A in the frontal cortex (right), but decreased 5-HT3A in the amygdala (left) and hypothalamus. GEG supplementation significantly increased 5-HT3A in the amygdala (left) and hypothalamus of treated SNL rats. The untreated SNL group had also decreased 5-HT2A in the frontal cortex (right), amygdala (right), and hippocampus (right). Administration of GEG to SNL rats significantly increased 5-HT2A in the amygdala (right) and hippocampus (left) compared to the untreated SNL group.

### 2.5. Neurotoxicity

**Figure 5:**
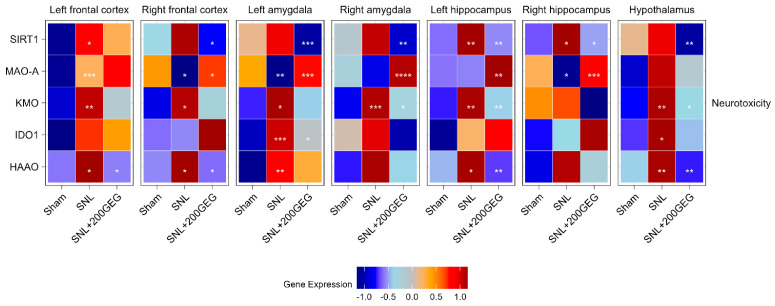
Effect of GEG supplementation on neurotoxicity mRNA expression. N = 6–8 per group. Data were analyzed by one-way ANOVA followed by the *post hoc* Tukey test at each time point for sham vs. SNL and SNL vs. SNL+200GEG for the respective gene. * *p* < 0.05. ** *p* < 0.01. *** *p* < 0.001. **** *p* < 0.0001.

Figure 5 illustrates the impact of GEG supplementation on neurotoxicity markers, namely, the mRNA SIRT1, MAOA, KMO, IDO1, and HAAO. The SNL group had increased SIRT1 in the frontal cortex (left) and hippocampus (both sides) relative to the sham group. GEG decreased SIRT1 in the frontal cortex (right), amygdala (both sides), hippocampus (both sides), and hypothalamus compared to the untreated SNL group. Relative to the sham group, GEG decreased MAOA in the frontal cortex (right), amygdala (left), and hippocampus (right) of the SNL group, while it increased MAOA in the frontal cortex (left). GEG increased MAOA in the frontal cortex (right), amygdala (both sides), and hippocampus (both sides) compared to the untreated SNL group. Compared to the sham group, the SNL group had increased KMO in all measured brain regions except for the hippocampus (right). GEG administration significantly suppressed KMO in the amygdala (right), hippocampus (left), and hypothalamus of SNL rats. The SNL group had increased IDO1 in the amygdala (left) and hypothalamus relative to the sham group. GEG administration in SNL rats decreased IDO1 in the amygdala (left). Compared to the sham group, the SNL group had increased HAAO in the frontal cortex (both sides), amygdala (left), hippocampus (left), and hypothalamus. GEG also suppressed HAAO in the frontal cortex (both sides), hippocampus (left), and hypothalamus of SNL rats, while SNL rats without GEG exhibited no such effects.

### 2.6. Correlation Between Behavior and Colonic Microbiome

**Figure 6:**
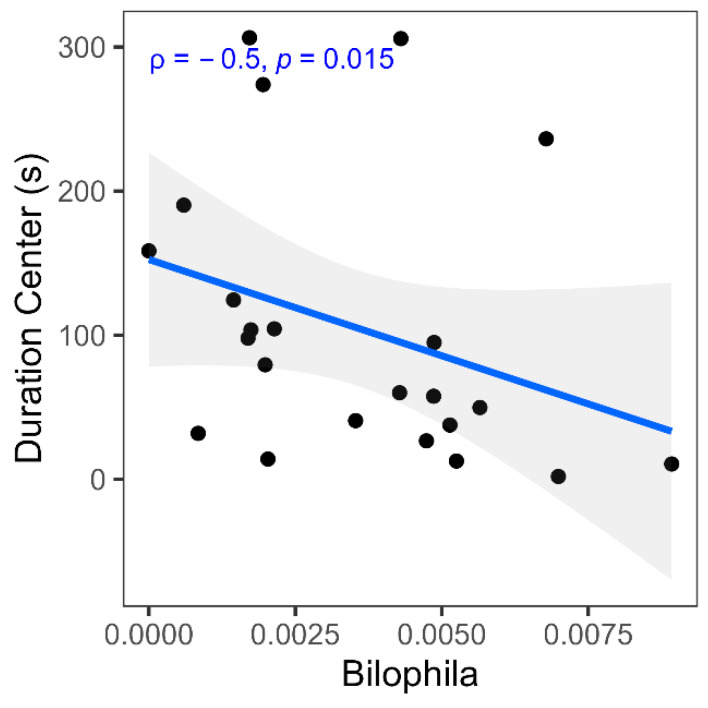
Spearman correlation between gut microbiome species and behavior measures. Scatter plot shows correlation coefficients (Spearman ρ) and *p*-value between relative abundance of *Bilophila* and duration center (s).

Figure 6 illustrates that the increased relative abundance of *Bilophila wadsworthia* correlates positively with decreased time spent in the open arms, suggesting that increased *Bilophila wadsworthia* abundance may have anxiolytic behavioral effects within the neuroinflammatory context of neuropathic pain.

## 3. Discussion

According to existing literature, this study presents the first report that GEG mitigates anxiety-like behavior in the context of neuropathic pain and identifies a coordinated set of molecular, metabolic, and microbiome changes that may underlie effects of GEG. Such findings corroborate previous studies that administration of ginger bioactive compounds has been shown to (i) decrease anxiety markers in the open field test as shown by increased center entry duration and frequency and (ii) mitigate emotional response to pain, such as audible and ultrasonic vocalization, in male SNL rats [23]. Moreover, ginger extracts were found to reduce anxio-depressive phenotypes in restraint stress [29], traumatic brain injury [26], diabetes mellitus [30], and electromagnetic field exposure [31]. Similarly, 6-shogaol, a major GEG bioactive component, has demonstrated the capacity to improve motor function and affective symptoms in mice with Parkinson disease [32], while red ginger extract mitigated acute anxiety behaviors by suppressing inflammation [33]. These anxiolytic-like effects appear to involve modulation of the serotonergic system, including activation of 5-HT1A receptors and antagonism of 5-HT3 receptors [23]. At the molecular level, ginger has been shown to significantly downregulate proinflammatory markers such as nuclear factor kappa-light-chain-enhancer of activated B cells (NF-κB), TNF-α, and IL-1β in the amygdala, colon, and other brain regions, while also reducing glial activation markers like glial fibrillary acidic protein and IBA1/CD11b in the brain and spinal cord of SNL-treated rats [34]. Collectively, these data suggest that the bioactive compounds of ginger act on converging pathophysiological processes which are shared across stress, injury, and metabolic disorders, particularly in the context of neuroinflammation, oxidative stress, disrupted neurotransmission, and colonic dysbiosis, all of which are core components of neuropathic pain and its anxio-depressive sequelae within the inflammatory continuum [26,35,36,37,38,39].

In this study, GEG produced robust, brain region-dependent molecular changes, spanning neuroplasticity, neurotransmission, neuroimmunity, and neurotoxicity pathways. Across the frontal cortex, amygdala, hippocampus, and hypothalamus, GEG counteracted SNL-induced alterations both in the direction and magnitude of gene expression. Collectively, these changes suggest that GEG restores homeostatic molecular states within cortico-limbic structures critical for affective regulation in pain and emotion. In addition, we also observed that GEG increased mRNA expression of NRF2, LXRα, and CX3CR1, particularly in the amygdala and hippocampus, which suggests that its anxiolytic actions are mediated by reinforcing antioxidant capacity, restoring lipid-based synaptic signaling, and normalizing microglial communication. These findings are consistent with prior reports that ginger bioactive compounds activate NRF2 signaling [40,41] and upregulate LXRα in metabolic tissues [42], reinforcing the translational relevance of these targets in the CNS.

Affective disturbances in neuropathic pain are closely tied to neuroinflammatory cascades. Proinflammatory cytokines released in the brain promote oxidative stress, impair monoamine synthesis, and aberrantly activate microglia, thereby causing a convergence of nociceptive input with mood dysregulation and stress excitotoxicity [37,39]. In this context, the focus on NRF2, LXRα, and CX3CR1 provides a targeted window into immune–neural interactions. Under normal conditions, NRF2 is often bound to, and polyubiquitinated by, KEAP1 in the cytoplasm for eventual proteasomal degradation. A transcription factor, NRF2 governs the antioxidant response, protects against oxidative damage, and regulates neurotransmitter balance; its disruption leads to both increased inflammation and anxio-depressive-like behaviors, whereas its activation enhances physiological resilience [36]. Best known as a regulator of cholesterol metabolism, LXRα forms a heterodimer with retinoid X receptor and influences membrane fluidity, synaptogenesis, and microglial polarization. By suppressing inflammatory microglial states and promoting hippocampal plasticity, LXRα activation exerts antidepressant- and anxiolytic-like effects [35,38,43]. CX3CR1, a G-protein-coupled receptor expressed on microglia, mediates neuron–microglia communication and ensures proper synaptic pruning; its downregulation heightens stress sensitivity and impairs synaptic plasticity [44,45]. Thus, the concomitant regulation of NRF2, LXRα, and CX3CR1 by GEG identifies a neuroimmune pathway with potential therapeutic relevance and sets the stage for the subsequent examination of broader molecular and metabolic adaptations.

Neuroinflammation and neuroplasticity are profoundly interconnected where prolonged inflammation can impair synaptic remodeling, especially in hippocampal and prefrontal circuits that govern mood, cognition, and stress responses [46,47,48]. For this reason, a focus on genes that bridge energy metabolism with neuroplasticity was paramount for this study, since metabolic reprogramming of glia is a hallmark of neuroinflammation. Within neuroplasticity-related pathways, in this study, we showed GEG regulated the mRNA PGK1, LDHA, MEK1, GPM6A, GLUT1, and HIF1α in a manner that varied by region. The most consistent restorative effects were observed in the frontal cortex, amygdala, and hippocampus—areas strongly linked to emotional regulation, stress integration, and memory encoding [49,50,51,52,53,54]. Each of these genes has a distinct but related role. These mRNA expression support neuronal metabolic resilience, mitochondrial function, synaptic remodeling, and neurite growth [55]. PGK1 and LDHA sustain glycolytic energy production; in neurons, these enzymes not only generate adenosine triphosphate (ATP) but also provide lactate as a signaling molecule that enhances plasticity [56,57,58]. MEK1, which is part of the central MAPK/ERK cascade, regulates neuronal differentiation and synaptic plasticity, while its downregulation has been linked to anxio-depressive-like behaviors [59]. Meanwhile, GPM6A is a glycoprotein essential for neurite outgrowth and synaptic stability, which is suppressed under chronic stress [60,61]. As the primary glucose transporter at the blood–brain barrier and astrocytes, GLUT1 ensures fuel availability for neurons engaged in energy-intensive synaptic remodeling [62]. Conversely, GEG suppressed HIF1α, which is elevated under chronic inflammatory or hypoxic stress and impairs synaptic function [63]. In the current study, the coordinated changes we observed suggest that GEG shifts brain energy metabolism away from inflammatory glycolysis and maladaptive hypoxia toward a state that supports neuronal resilience and plasticity. Taken together, these region-specific responses point to the ability of GEG to promote neuroplastic adaptations in networks most closely associated with anxiety-like behavior.

GEG also orchestrated coordinated neurotransmission changes that were regionally distributed across limbic and cortical structures. Disruptions in neurotransmission are a defining feature of anxiety and depression, particularly within serotonergic and glutamatergic systems. Examining the mRNA SLC1A2, SLC1A3, GRM5, GRIN2B, GRIN2C, GRIA1, CHRNA7, 5-HT2A, and 5-HT3A offers insight into the neurotransmission systems implicated in NP-related anxiety. Our findings indicate that GEG modulates several critical nodes within these networks. Upregulation of SLC1A2 and SLC1A3 in the frontal cortex suggested enhanced glutamate clearance through excitatory amino acid transporter (EAAT) 1/2, thereby reducing excitotoxic risk; this is notable since decreased EAAT expression has been documented in cortical regions of depressed patients, correlating with synaptic dysfunction [64,65]. In the hippocampus and hypothalamus, GEG increased expression of GRM5, a receptor essential for cortico-limbic circuit integrity and pain modulation, with augmented mGluR5 translating to increased glutamate signaling, whose downregulation has been linked to stress vulnerability and anxiety [66,67,68]. Consistent with this, GEG also attenuated expression of excitotoxic N-methyl-D-aspartate (NMDA) receptor subunits GRIN2B and GRIN2C in the frontal cortex and hypothalamus, thereby reflecting impaired neuroplasticity and neurotoxicity that have been implicated in pain hypersensitivity and anxiety [69,70]. By contrast, AMPA receptors such as GRIA1, which support fast excitatory glutamatergic neurotransmission and synaptic plasticity, were downregulated under GEG treatment, suggesting selective targeting of excitotoxic NMDA-driven mechanisms rather than suppression of physiological glutamatergic tone [71]. These adjustments are complemented by increased expression of 5-HT3A receptor in the amygdala and hypothalamus, implying improved serotonergic tone. 5-HT3A is known to mediate anxiolytic and anti-depressant responses [72]. Finally, GEG suppressed expression of CHRNA7, which may reduce cholinergic-driven neuroimmune activation as well as cognitive and affective processes [73] across several brain regions. The convergence of these molecular shifts, especially in the amygdala and hippocampus, aligns with anxiolytic behavioral outcomes [49,50,51].

Beyond glutamatergic regulation, GEG influenced serotonergic pathways by downregulating IDO1 and MAOA, thereby limiting tryptophan diversion into the kynurenine pathway and slowing monoamine degradation, respectively. Our findings indicate that GEG does not act on a single neurotransmitter pathway but instead orchestrates a broad rebalancing of glutamatergic, serotonergic, and cholinergic systems. By reducing excitotoxic drive, preserving monoamine tone, and strengthening receptor signaling in cortico-limbic circuits, GEG mitigates the neurotransmission of disturbances that underlie anxiety-like behaviors in neuropathic pain. Nonetheless, inflammation concretely drives neurotoxicity through the kynurenine pathway. When proinflammatory cytokines (e.g., TNF-α, IL-6) arrive through the vasa nervorum, these molecules activate IDO1 in the arterial endothelial cells, which then initiates tryptophan catabolism via the kynurenine, JAK/STAT, and non-canonical NF-κB pathways [74,75]. Such downstream cascades can lead to accumulation of neurotoxic metabolites like quinolinic acid and 3-hydroxykynurenine produced by KMO and HAAO proteins, inducing potent NMDA receptor agonism, during upregulation of NAD^+^ *de novo* synthesis. Moreover, higher concentrations of NAD^+^ lead to increased activity of the NAD^+^-dependent deacetylase SIRT1, which is not only critical for epigenetic modification but also functions as a transcription factor in many physiological processes [76]. Suppression of SIRT1 is noteworthy in that elevated levels of activity enhance MAOA expression and accelerate serotonin turnover, promoting anxiety-like behaviors [77,78]. Furthermore, excess quinolinic acid overstimulates both GRIN2B and GRIN2C subunits, inducing calcium overload and mitochondrial dysfunction [79,80]. Consistent with clinical observations of receptor upregulation in depressed brains [81,82], this pathway links immune activation to excitotoxicity. In this study, GEG suppressed neurotoxic and neuroimmune-activating genes (i.e., SIRT1, IDO1, KMO, HAAO), especially in the amygdala, hippocampus, and hypothalamus, suggesting reduced quinolinic acid and 3-hydroxykynurenine production as well as preserved tryptophan pools for serotonin synthesis. The dual function of GEG in dampening glutamatergic excitotoxic drive and preserving monoamine tone is promising.

It is important to note that all neuroimmunity-, neuroplasticity-, neurotransmission-, and neurotoxicity-related gene assessments were performed at the level of discrete brain regions. This approach revealed that GEG does not induce global or uniform molecular changes, but rather modulates gene expression in a region-specific manner. Particularly, the frontal cortex, amygdala, hippocampus, and hypothalamus exhibited distinct patterns of GEG-dependent responses that align with the known role of each region in emotional processing, pain modulation, and stress integration.

The intestinal microbiota also emerged as a critical upstream regulator of neuroimmune and neurobehavioral processes. Dysbiosis alters microbial community composition, favoring pathogenic or opportunistic species over beneficial commensals, disrupts the intestinal epithelial barrier, and promotes the subsequent translocation of bacterial products such as lipopolysaccharides (LPS) into circulation (i.e., antigenemia). These products trigger systemic cytokine release, which is able to permeate the blood–brain barrier (BBB) and drive microglial activation, suggesting peripheral immune activation is integral CNS inflammation [83,84]. Among dysbiotic taxa, *Bilophila wadsworthia* is particularly notable, as it has been associated with bile acid dysmetabolism, impaired epithelial barrier integrity, and heightened activation of the kynurenine pathway through IDO1 induction [85,86]. In the present study, *Bilophila* relative abundance was positively correlated with anxiety-like behaviors, reinforcing the mechanistic link between colonic dysbiosis, tryptophan diversion away from serotonin synthesis, and NMDA receptor–mediated neuroinflammation. Such a positive correlation between *Bilophila* relative abundance and anxiety-like behavior is supported by a case–control study showing increased *Bilophila* abundance in patients with major depressive disorder than in the healthy controls [87]. On the other hand, *Bilophila* has been linked to proinflammatory responses, which can promote the expansion of proinflammatory T helper type 1 (Th1) cells, resulting in the production of interferon-gamma (IFN-γ) in the gut. This immune response can lead to anxiety and cognitive impairment, as IFN-γ is associated with detrimental effects on the brain [88]. The immunological inflammatory response is a contributing factor in the development of anxiety and depression in humans [89] and animals [90]. Studies of individuals with clinically diagnosed anxiety have shown higher levels of proinflammatory cytokines than healthy individuals [91]. These findings suggest that part of the therapeutic benefit of GEG arises from modulation of the gut–brain–immune axis, especially Th1-leaning, attenuating dysbiosis-driven signaling cascades that connect peripheral microbial changes with central affective outcomes.

The present study has several limitations. All experiments were conducted in male rats, limiting generalizability between the sexes. Furthermore, while murine models provide valuable mechanistic insights, they cannot fully replicate the complexity of human psychiatric disorders. The relatively small sample size and reliance on mRNA expression without confirmatory proteomics also constrain the interpretability of our findings. Moreover, although BDNF is a widely accepted and used marker of synaptic plasticity, we felt downstream regulators such as PGK1 and GPM6A provide a more direct window into the metabolic and structural plasticity mechanisms most relevant to our study aims.

While microbiota modulation (i.e., *Bilophila* relative abundance) via GEG may contribute to transcriptional alterations in different brain regions, the positive correlation found in this study does not establish a causal relationship between the variables, necessitating further studies. We noted that the study in its cross-sectional design does not address temporal dynamics of gene expression or microbiome shifts over the disease course and that the behavioral conclusion is based on a single behavioral paradigm, which could decrease its robustness. With the limited availability of brain tissues, we were not able to incorporate immunohistochemical analyses of microglial activation and neuronal survival markers in the collected brain tissues to compensate for the limited behavioral testing and provide biological validation of the proposed neuroprotective or anti-inflammatory effects of GEG in the present study. A future animal study with a larger sample size should include such immunohistochemical validation of the brain tissues. Despite these limitations, the integration of behavioral, molecular, and microbiome data supports the therapeutic potential of GEG and emphasizes the gut–brain–immune axis as a promising target for future translational work.

## 4. Materials and Methods

### 4.1. Animals, Neuropathic Pain Induction, and Group Treatments

The Institutional Animal Care and Use Committee (IACUC) at Texas Tech University Health Sciences Center (protocol number 20023) approved all procedures of this animal on 31 January 2021. We purchased twenty-seven male Sprague Dawley rats from Envigo, Cumberland, VA, USA and kept them individually housed in a 12 h light/12 h dark cycle throughout the study period. After 5 days of acclimation, we performed a sham procedure and another 18 rats received an SNL procedure. The detailed procedures of sham and SNL surgeries are described in our previously published studies [92,93].

The animals in the sham group and in the SNL group were given vehicle corn oil daily via oral gavage for 5 weeks. The animals in the SNL+200GEG group received the SNL procedure and were given GEG at 200 mg/kg body weight (BW) daily via oral gavage for 5 weeks. GEG was composed of 6-gingerol (18.7%), 8-gingerol (1.81%), 10-gingerol (2.86%), 6-shogoal (3.09%), 8-shogaol (0.39%), and 10-shogaol (0.41%). All animals were fed with the AIN-93G diet (Research Diet Inc., New Brunswick, NJ, USA) and water ad libitum throughout the study period. Measurements of food intake, water consumption, and body weight were taken on a weekly basis.

We performed a power analysis based on preliminary data and previous studies from our laboratory [94]. To detect a significant change in anxiety-like behavior at α = 0.05 with 90% power, a sample size of 8–10 rats per group was considered sufficient. Thus, we used n = 8–10 rats per group to ensure adequate statistical power.

### 4.2. Anxiety-like Behavior Outcome Measurement

Before conducting surgery and final sample collection, we measured avoidance behavior as an outcome measure of anxiety-like behavior using the EPM based on our previously published work [95]. We record how many entries the rats made and their duration in the open arms during the first 5 minutes. To ensure consistent behavioral testing, the same person carried out all assessments but was blinded to the group assignment. Test conditions (time of day, environment, and place) were kept uniform throughout the study.

### 4.3. Sample Collection

At the end of the study, the animals were anesthetized under isoflurane and euthanized for sample collection. The frontal cortex (both sides, medial prefrontal cortex), amygdala (both sides, central nucleus), hippocampus (both sides, dorsal), hypothalamus, and feces from the cecum were harvested between 11 am and 4 pm of the day with group rotation to avoid time-effect. Samples were snapped in liquid nitrogen and stored at −80 °C for later laboratory analyses.

### 4.4. mRNA Gene Expresison via qRT-PCR

We extracted total RNA from the frontal cortex, amygdala, hippocampus, and hypothalamus, then transcribed into complementary DNA (cDNA) to amplify the targeted genes with respective primers (see Supplement Table S1 for a list of primer sets) according to our previous work [14,27]. We conducted qRT-PCR to amplify the targeted gene expression level according to the primary function for the context of this study. The first encompassed neuroinflammation/neuroimmunity-associated genes comprising nuclear factor erythroid 2-related factor 2 (NRF2), liver X receptor alpha (LXRα), and CX3C motif chemokine receptor 1 (CX3CR1). The second explored neuroplasticity-associated genes that included phosphoglycerate kinase 1 (PGK1), mitogen-activated protein kinase kinase 1 (MEK1), lactate dehydrogenase A (LDHA), hypoxia-inducible factor 1 alpha (HIF1α), glycoprotein M6A (GPM6A), and glucose transporter protein type 1 (GLUT1). The third involved neurotransmission-associated genes including solute carrier family 1 member 3 (SLC1A3), solute carrier family 1 member 2 (SLC1A2), metabotropic glutamate receptor 5 (GRM5), glutamate ionotropic receptor NMDA type subunit 2C (GRIN2C) and 2B (GRIN2B), glutamate ionotropic receptor AMPA type subunit 1 (GRIA1), alpha-7 nicotinic acetylcholine receptor (α7nAChR) subunit (CHRNA7), subunit A of the 5-hydroxytryptamine receptor 3 (5-HT3A), and subunit A of the 5-hydroxytryptamine receptor 2 (5-HT2A). The fourth and final panel comprised neurotoxicity-associated genes, including monoamine oxidase A (MAOA), sirtuin-1 (SIRT1), indoleamine 2,3-dioxygenase (IDO1), kynurenine 3-monooxygenase (KMO), and 3-hydroxyanthranilate 3,4-dioxygenase (HAAO). We used corresponding primers (Supplement Table S1) based on our previously published methods [28,96]. We normalized all gene expression levels to a control of β-actinand used the formula x = 2 − (ΔCT × 1000) to calculate the level of gene expression [97].

### 4.5. Relative Abundance of Bilophila

The relative abundance of *Bilophila* was obtained from the previously analyzed microbiome dataset from the same animals as described in the introduction [28]. Gut microbiota profiling was performed via 16S rRNA amplicon sequencing by isolating fecal DNA using the PowerFecal DNA isolation kit and amplicon sequencing the V4 variable regions of the 16S rRNA using PCR primer 515F/806R.

### 4.6. Statistical Analysis

For EPM data, we performed a one-way ANOVA followed by the post hoc Tukey test at each time point to assess statistical significance. We analyzed gene expression differences with one-way ANOVA followed by the post hoc Tukey test for multiple comparisons. We conducted a secondary analysis using the Spearman rank correlation to assess the relationship between EPM parameters and colonic microbiome composition. * *p* < 0.05, ** *p* <0.01, *** *p* < 0.001, **** *p* < 0.0001.

## 5. Conclusions

These multifaceted analyses of gene expression within the context of neuroinflammation, neuroimmunity, neuroplasticity, neurotransmission, and neurotoxicity highlight the complex pathophysiology underlying anxiety-like states and their mitigation by GEG. Many of these pathways converge on glial immune signaling, metabolic shifts, and neurotransmitter regulation, providing a compelling rationale for integrative therapies that target upstream modulators such as the gut microbiome. These findings suggest that GEG influences these interconnected networks, in part through modulation of the gut–brain axis, offering a potential disease-modifying adjuvant approach in the management of anxiety and related psychiatric disorders. An intriguing observation was the downregulation of GRM5 in the right amygdala, which contrasts with prior reports of mGluR5 upregulation in pain models and may reflect adaptive responses to chronicity in this neuropathic pain paradigm. Such findings underscore the dynamic and region-specific nature of neurotransmitter regulation in affective states. 

## Data Availability

The raw sequencing data (BioProject access number PRJNA935472) of 16S rRNA amplicon sequencing were deposited in the National Center for Biotechnology Information BioProject database. The original contributions presented in this study are included in the article. Further inquiries can be directed to the corresponding authors.

## Supplementary Materials

The following supporting information can be downloaded at https://www.mdpi.com/article/10.3390/molecules31010166/s1, Table S1: List of primers for mRNA.

## Abbreviations

IBA1: ionized calcium-binding adapter molecule 1; CD11b: cluster of differentiation 11b; KEAP1: Kelch-like ECH-associated protein 1; MAPK/ERK: mitogen-activated protein kinase/extracellular signal-regulated kinase; AMPA: α-amino-3-hydroxy-5-methyl-4-isoxazolepropionic acid.

## References

[1] Martins J.P., Marson F.A.L. (2024). A narrative review of the complex panorama regarding chronic neuropathic pain mainly for the psychological issues. Heliyon.

[2] School H.M. (2007). National Comorbidity Survey (NCS). https://www.hcp.med.harvard.edu/ncs/index.php.

[3] (2020). The Lancet Global Health. Mental health matters. Lancet Glob. Health.

[4] Greenberg P.E., Fournier A.A., Sisitsky T., Simes M., Berman R., Koenigsberg S.H., Kessler R.C. (2021). The Economic Burden of Adults with Major Depressive Disorder in the United States (2010 and 2018). Pharmacoeconomics.

[5] Jesulola E., Micalos P., Baguley I.J. (2018). Understanding the pathophysiology of depression: From monoamines to the neurogenesis hypothesis model—Are we there yet?. Behav. Brain Res..

[6] Mariani N., Cattane N., Pariante C., Cattaneo A. (2021). Gene expression studies in Depression development and treatment: An overview of the underlying molecular mechanisms and biological processes to identify biomarkers. Transl. Psychiatry.

[7] Bhatt S., Nagappa A.N., Patil C.R. (2020). Role of oxidative stress in depression. Drug Discov. Today.

[8] Suneson K., Lindahl J., Chamli Harsmar S., Soderberg G., Lindqvist D. (2021). Inflammatory Depression-Mechanisms and Non-Pharmacological Interventions. Int. J. Mol. Sci..

[9] Menke A. (2019). Is the HPA Axis as Target for Depression Outdated, or Is There a New Hope?. Front. Psychiatry.

[10] Varghese F.P., Brown E.S. (2001). The Hypothalamic-Pituitary-Adrenal Axis in Major Depressive Disorder: A Brief Primer for Primary Care Physicians. Prim. Care Companion J. Clin. Psychiatry.

[11] Otte C., Wingenfeld K., Kuehl L.K., Kaczmarczyk M., Richter S., Quante A., Regen F., Bajbouj M., Zimmermann-Viehoff F., Wiedemann K. (2015). Mineralocorticoid Receptor Stimulation Improves Cognitive Function and Decreases Cortisol Secretion in Depressed Patients and Healthy Individuals. Neuropsychopharmacology.

[12] Leng F.D., Edison P. (2021). Neuroinflammation and microglial activation in Alzheimer disease: Where do we go from here?. Nat. Rev. Neurol..

[13] Li Q.Y., Barres B.A. (2018). Microglia and macrophages in brain homeostasis and disease. Nat. Rev. Immunol..

[14] Wang R., Santos J.M., Dufour J.M., Stephens E.R., Miranda J.M., Washburn R.L., Hibler T., Kaur G., Lin D., Shen C.L. (2022). Ginger Root Extract Improves GI Health in Diabetic Rats by Improving Intestinal Integrity and Mitochondrial Function. Nutrients.

[15] Evrensel A., Ünsalver B.Ö., Ceylan M.E. (2020). Neuroinflammation, Gut-Brain Axis and Depression. Psychiatry Investig..

[16] Chevalier G., Siopi E., Guenin-Macé L., Pascal M., Laval T., Rifflet A., Boneca I.G., Demangel C., Colsch B., Pruvost A. (2020). Effect of gut microbiota on depressive-like behaviors in mice is mediated by the endocannabinoid system. Nat. Commun..

[17] Irum N., Afzal T., Faraz M.H., Aslam Z., Rasheed F. (2023). The role of gut microbiota in depression: An analysis of the gut-brain axis. Front. Behav. Neurosci..

[18] Lin P., Li D., Shi Y., Li Q.T., Guo X.K., Dong K., Chen Q., Lou X., Li Z., Li P. (2023). Dysbiosis of the Gut Microbiota and Kynurenine (Kyn) Pathway Activity as Potential Biomarkers in Patients with Major Depressive Disorder. Nutrients.

[19] Zhu Z., Cheng Y., Liu X., Xu X., Ding W., Ling Z., Liu J., Cai G. (2025). The microbiota-gut-brain axis in depression: Unraveling the relationships and therapeutic opportunities. Front. Immunol..

[20] Hao W.Z., Ma Q.Y., Wang L., Yuan N.J., Gan H., He L.L., Li X., Huang J., Chen J. (2024). Gut dysbiosis induces the development of depression-like behavior through abnormal synapse pruning in microglia-mediated by complement C3. Microbiome.

[21] Liaqat H., Parveen A., Kim S.Y. (2022). Neuroprotective Natural Products’ Regulatory Effects on Depression via Gut-Brain Axis Targeting Tryptophan. Nutrients.

[22] O’Riordan K.J., Moloney G.M., Keane L., Clarke G., Cryan J.F. (2025). The gut microbiota-immune-brain axis: Therapeutic implications. Cell. Rep. Med..

[23] Shen C.L., Wang R., Yakhnitsa V., Santos J.M., Watson C., Kiritoshi T., Ji G., Hamood A.N., Neugebauer V. (2022). Gingerol-Enriched Ginger Supplementation Mitigates Neuropathic Pain via Mitigating Intestinal Permeability and Neuroinflammation: Gut-Brain Connection. Front. Pharmacol..

[24] Yazdi G.M., Vaezi G., Hojati V., Mohammad-Zadeh M. (2021). The Effect of 6-gingerol on Growth Factors and Apoptosis Indices in Rats Exposed to Gold Nanoparticles. Basic. Clin. Neurosci..

[25] Bano S., Sharif H., Badawy A.A. (2021). Effects of oral administration of an aqueous ginger extract on anxiety behavior and tryptophan and serotonin metabolism in the rat. Asian J. Med. Sci..

[26] Afzal M., Kazmi I., Quazi A.M., Khan S.A., Zafar A., Al-Abbasi F.A., Imam F., Alharbi K.S., Alzarea S.I., Yadav N. (2022). 6-Shogaol Attenuates Traumatic Brain Injury-Induced Anxiety/Depression-like Behavior via Inhibition of Oxidative Stress-Influenced Expressions of Inflammatory Mediators TNF-α, IL-1β, and BDNF: Insight into the Mechanism. Acs Omega.

[27] Shen C.L., Wang R., Santos J.M., Elmassry M.M., Stephens E., Kim N., Neugebauer V. (2024). Ginger alleviates mechanical hypersensitivity and anxio-depressive behavior in rats with diabetic neuropathy through beneficial actions on gut microbiome composition, mitochondria, and neuroimmune cells of colon and spinal cord. Nutr. Res..

[28] Shen C.L., Santos J.M., Elmassry M.M., Bhakta V., Driver Z., Ji G.C., Yakhnitsa V., Kiritoshi T., Lovett J., Hamood A.N. (2024). Ginger Polyphenols Reverse Molecular Signature of Amygdala Neuroimmune Signaling and Modulate Microbiome in Male Rats with Neuropathic Pain: Evidence for Microbiota-Gut-Brain Axis. Antioxidants.

[29] Upadhyaya K., Sharma P.K., Akhtar A., Pilkhwal Sah S. (2022). Protective Effects of Zingerone Against Depression-Like Behavior and Biochemical Changes in Chronic Stressed Rats: Antioxidant Effects. J. Med. Food.

[30] Farzin D., Fathiazad F., Fazellian M. (2013). Antidepressant effect of methanolic ginger extract in diabetic mice using forced-swim test. J. Maz. Univ. Med. Sci..

[31] Khaki A., Farnam A., Rouhani S., Imantalab B., Seery S. (2013). Androgenic activity evaluation of ginger rhizome in reducing depression in the forced swimming test of rats Exposed to Electromagnetic Field (EMF). Int. J. Women’s Heal. Reprod. Sci..

[32] Kim J.H., Yoon H.J., Choi Y., Kim J.S., Ju I.G., Eo H., Lee S., Cho J.-Y., Park B.Y., Hong S.-P. (2025). 6-Shogaol, a neuro-nutraceutical derived from ginger, alleviates motor symptoms and depression-like behaviors and modulates the release of monoamine neurotransmitters in Parkinson’s disease mice. Eur. J. Nutr..

[33] Dania I.A., Rambe A.S., Harahap U., Effendy E., Wahmurti T., Ilyas S., Rusda M., Amin M.M. (2023). Red Ginger’s Anti-Anxiety Effect on BALB/c Strain Mice (*Mus musculus*) Pro-Inflammatory and Anti-Inflammatory Measurements as Anxiety Model. Baghdad Sci. J..

[34] Shen C.L., Santos J.M., Kiritoshi T., Guangchen J., Presto P., Yakhnitsa V., Antenucci N., Lovett J., Neugebauer V. (2024). Effects of ginger supplementation on neuroinflammation and mitochondrial function in spinal cords of rats with neuropathic pain: Evidence for sex differences. Curr. Dev. Nutr..

[35] Khairova R.A., Machado-Vieira R., Du J., Manji H.K. (2009). A potential role for pro-inflammatory cytokines in regulating synaptic plasticity in major depressive disorder. Int. J. Neuropsychopharmacol..

[36] Martin-de-Saavedra M.D., Budni J., Cunha M.P., Gómez-Rangel V., Lorrio S., del Barrio L., Lastres-Becker I., Parada E., Tordera R.M., Rodrigues A.L.S. (2013). Nrf2 participates in depressive disorders through an anti-inflammatory mechanism. Psychoneuroendocrinology.

[37] Salcudean A., Popovici R.A., Pitic D.E., Sârbu D., Boroghina A., Jomaa M., Salehi M.A., Kher A.A.M., Lica M.M., Bodo C.R. (2025). Unraveling the Complex Interplay Between Neuroinflammation and Depression: A Comprehensive Review. Int. J. Mol. Sci..

[38] Sandoval-Hernández A.G., Buitrago L., Moreno H., Cardona-Gómez G.P., Arboleda G. (2015). Role of Liver X Receptor in AD Pathophysiology. PLoS ONE.

[39] Shen C.L., Hassan T., Presto P., Payberah D., Devega R., Wakefield S., Dunn D.M., Neugebauer V. (2025). Novel Insights into Dietary Bioactive Compounds and Major Depressive Disorders: Evidence from Animal Studies and Future Perspectives. J. Nutr..

[40] Chen H.D., Fu J.S., Chen H., Hu Y.H., Soroka D.N., Prigge J.R., Schmidt E.E., Yan F., Major M.B., Chen X. (2014). Ginger Compound [6]-Shogaol and Its Cysteine-Conjugated Metabolite (M2) Activate Nrf2 in Colon Epithelial Cells in Vitro and in Vivo. Chem. Res. Toxicol..

[41] Peng S., Yao J., Liu Y., Duan D., Zhang X., Fang J. (2015). Activation of Nrf2 target enzymes conferring protection against oxidative stress in PC12 cells by ginger principal constituent 6-shogaol. Food Funct..

[42] Dixon E.D., Nardo A.D., Claudel T., Trauner M. (2021). The role of lipid sensing nuclear receptors (PPARs and LXR) and metabolic lipases in obesity, diabetes and NAFLD. Genes.

[43] Xu X.X., Xiao X., Yan Y.X., Zhang T. (2021). Activation of liver X receptors prevents emotional and cognitive dysfunction by suppressing microglial M1-polarization and restoring synaptic plasticity in the hippocampus of mice. Brain Behav. Immun..

[44] Corona A.W., Huang Y., O’Connor J.C., Dantzer R., Kelley K.W., Popovich P.G., Godbout J.P. (2010). Fractalkine receptor (CXCR1) deficiency sensitizes mice to the behavioral changes induced by lipopolysaccharide. J. Neuroinflamm..

[45] Hoshiko M., Arnoux I., Avignone E., Yamamoto N., Audinat E. (2012). Deficiency of the Microglial Receptor CX3CR1 Impairs Postnatal Functional Development of Thalamocortical Synapses in the Barrel Cortex. J. Neurosci..

[46] Calderone A., Latella D., Cardile D., Gangemi A., Corallo F., Rifici C., Quartarone A., Calabrò R.S. (2024). The Role of Neuroinflammation in Shaping Neuroplasticity and Recovery Outcomes Following Traumatic Brain Injury: A Systematic Review. Int. J. Mol. Sci..

[47] Price R.B., Duman R. (2020). Neuroplasticity in cognitive and psychological mechanisms of depression: An integrative model. Mol. Psychiatry.

[48] Radulescu I., Dragoi A.M., Trifu S.C., Cristea M.B. (2021). Neuroplasticity and depression: Rewiring the brain’s networks through pharmacological therapy (Review). Exp. Ther. Med..

[49] Dominguez-Borras J., Vuilleumier P. (2022). Amygdala function in emotion, cognition, and behavior. Handb. Clin. Neurol..

[50] LaBar K.S., Cabeza R. (2006). Cognitive neuroscience of emotional memory. Nat. Rev. Neurosci..

[51] Mainardi M., Fusco S., Grassi C. (2015). Modulation of hippocampal neural plasticity by glucose-related signaling. Neural Plast..

[52] Parvizi J., Veit M.J., Barbosa D.A.N., Kucyi A., Perry C., Parker J.J., Shivacharan R.S., Chen F., Yih J., Gross J.J. (2022). Complex negative emotions induced by electrical stimulation of the human hypothalamus. Brain Stimul..

[53] Shao L.X., Liao C., Gregg I., Davoudian P.A., Savalia N.K., Delagarza K., Kwan A.C. (2021). Psilocybin induces rapid and persistent growth of dendritic spines in frontal cortex in vivo. Neuron.

[54] Strobel C., Hunt S., Sullivan R., Sun J., Sah P. (2014). Emotional regulation of pain: The role of noradrenaline in the amygdala. Sci. China Life Sci..

[55] Chai W.W., Eaton S., Rasmussen H.E., Tao M.H. (2021). Associations of Dietary Lipid-Soluble Micronutrients with Hepatic Steatosis among Adults in the United States. Biomedicines.

[56] Benarroch E. (2024). What Is the Role of Lactate in Brain Metabolism, Plasticity, and Neurodegeneration?. Neurology.

[57] Kokotos A.C., Antoniazzi A.M., Unda S.R., Ko M.S., Park D., Eliezer D., Kaplitt M.G., De Camilli P., Ryan T.A. (2024). Phosphoglycerate kinase is a central leverage point in Parkinson’s disease-driven neuronal metabolic deficits. Sci. Adv..

[58] Liu Y.W., Zhong C.H., Yang Y.X., Hu J.B., Yi X.Y., Huang J.T., Li H., Liu X., Xue K., Chen X. (2025). The Role of Mitochondrial Energy Metabolism in the Mechanism of Exercise Improving Depression. Curr. Issues Mol. Biol..

[59] Wefers B., Hitz C., Hölter S.M., Trümbach D., Hansen J., Weber P., Pütz B., Deussing J.M., De Angelis M.H., Roenneberg T. (2012). MAPK Signaling Determines Anxiety in the Juvenile Mouse Brain but Depression-Like Behavior in Adults. PLoS ONE.

[60] Aparicio G.I., León A., Fuster R.G., Ravenscraft B., Monje P.V., Scorticati C. (2023). Endogenous Glycoprotein GPM6a Is Involved in Neurite Outgrowth in Rat Dorsal Root Ganglion Neurons. Biomolecules.

[61] Fuchsova B., Juliá A.A., Rizavi H.S., Frasch A.C., Pandey G.N. (2015). Altered Expression of Neuroplasticity-Related Genes in the Brain of Depressed Suicides. Neuroscience.

[62] Olson A.L., Pessin J.E. (1996). Structure, function, and regulation of the mammalian facilitative glucose transporter gene family. Annu. Rev. Nutr..

[63] Shibata T., Yamagata H., Uchida S., Otsuki K., Hobara T., Higuchi F., Abe N., Watanabe Y. (2013). The alteration of hypoxia inducible factor-1 (HIF-1) and its target genes in mood disorder patients. Prog. Neuropsychopharmacol. Biol. Psychiatry.

[64] Harvey B.K., Airavaara M., Hinzman J., Wires E.M., Chiocco M.J., Howard D.B., Shen H., Gerhardt G., Hoffer B.J., Wang Y. (2011). Targeted Over-Expression of Glutamate Transporter 1 (GLT-1) Reduces Ischemic Brain Injury in a Rat Model of Stroke. PLoS ONE.

[65] Rodek P., Kowalczyk M., Kowalski J., Owczarek A., Choreza P., Kucia K. (2022). Association Study of the SLC1A2 (rs4354668), SLC6A9 (rs2486001), and SLC6A5 (rs2000959) Polymorphisms in Major Depressive Disorder. J. Clin. Med..

[66] Deschwanden A., Karolewicz B., Feyissa A.M., Treyer V., Ametamey S.M., Johayem A., Burger C., Auberson Y.P., Sovago J., Stockmeier C.A. (2011). Reduced metabotropic glutamate receptor 5 density in major depression determined by [(11)C]ABP688 PET and postmortem study. Am. J. Psychiatry.

[67] Notartomaso S., Antenucci N., Mazzitelli M., Rovira X., Boccella S., Ricciardi F., Liberatore F., Gomez-Santacana X., Imbriglio T., Cannella M. (2024). A ‘double-edged’ role for type-5 metabotropic glutamate receptors in pain disclosed by light-sensitive drugs. eLife.

[68] Shin S., Kwon O., Kang J.I., Kwon S., Oh S., Choi J., Kim C.H., Kim D.G. (2015). mGluR5 in the nucleus accumbens is critical for promoting resilience to chronic stress. Nat. Neurosci..

[69] Kaur A., Singh L., Singh N., Bhatti M.S., Bhatti R. (2019). Ameliorative effect of imperatorin in chemically induced fibromyalgia: Role of NMDA/NFkB mediated downstream signaling. Biochem. Pharmacol..

[70] Zhou H.-Y., Chen S.-R., Pan H.-L. (2011). Targeting N-methyl-D-aspartate receptors for treatment of neuropathic pain. Expert. Rev. Clin. Pharmacol..

[71] Ismail V., Zachariassen L.G., Godwin A., Sahakian M., Ellard S., Stals K.L., Baple E., Brown K.T., Foulds N., Wheway G. (2022). Identification and functional evaluation of GRIA1 missense and truncation variants in individuals with ID: An emerging neurodevelopmental syndrome. Am. J. Hum. Genet..

[72] Chia J.S.M., Farouk A.A.O., Mohamad A.S., Sulaiman M.R., Perimal E.K. (2016). Zerumbone alleviates chronic constriction injury-induced allodynia and hyperalgesia through serotonin 5-HT receptors. Biomed. Pharmacother..

[73] Maroli A., Di Lascio S., Drufuca L., Cardani S., Setten E., Locati M., Fornasari D., Benfante R. (2019). Effect of donepezil on the expression and responsiveness to LPS of CHRNA7 and CHRFAM7A in macrophages: A possible link to the cholinergic anti-inflammatory pathway. J. Neuroimmunol..

[74] Forteza M.J., Polyzos K.A., Baumgartner R., Suur B.E., Mussbacher M., Johansson D.K., Hermansson A., Hansson G.K., Ketelhuth D.F.J. (2018). Activation of the Regulatory T-Cell/Indoleamine 2,3-Dioxygenase Axis Reduces Vascular Inflammation and Atherosclerosis in Hyperlipidemic Mice. Front. Immunol..

[75] Huang X.T., Zhang F., Wang X.B., Liu K. (2022). The Role of Indoleamine 2, 3-Dioxygenase 1 in Regulating Tumor Microenvironment. Cancers.

[76] Yamamoto H., Schoonjans K., Auwerx J. (2007). Sirtuin functions in health and disease. Mol. Endocrinol..

[77] Libert S., Pointer K., Bell E.L., Das A., Cohen D.E., Asara J.M., Kapur K., Bergmann S., Preisig M., Otowa T. (2011). SIRT1 Activates MAO-A in the Brain to Mediate Anxiety and Exploratory Drive. Cell.

[78] Naoi M., Riederer P., Maruyama W. (2016). Modulation of monoamine oxidase (MAO) expression in neuropsychiatric disorders: Genetic and environmental factors involved in type A MAO expression. J. Neural Transm..

[79] Hosoi R., Fujii Y., Hiroyuki O., Shukuri M., Nishiyama S., Kanazawa M., Todoroki K., Arano Y., Sakai T., Tsukada H. (2021). Evaluation of intracellular processes in quinolinic acid-induced brain damage by imaging reactive oxygen species generation and mitochondrial complex I activity. EJNMMI Res..

[80] Jhamandas K.H., Boegman R.J., Beninger R.J., Miranda A.F., Lipic K.A. (2000). Excitotoxicity of quinolinic acid: Modulation by endogenous antagonists. Neurotox. Res..

[81] Brown S.J., Brown A.M., Purves-Tyson T.D., Huang X.F., Weickert C.S., Newell K.A. (2023). gene expression is increased in the anterior cingulate cortex in major depression. J. Psychiatr. Res..

[82] Saez E., Erkoreka L., Moreno-Calle T., Berjano B., Gonzalez-Pinto A., Basterreche N., Arrue A. (2022). Genetic variables of the glutamatergic system associated with treatment-resistant depression: A review of the literature. World J. Psychiatry.

[83] Bercik P., Denou E., Collins J., Jackson W., Lu J., Jury J., Deng Y., Blennerhassett P., Macri J., McCoy K.D. (2011). The Intestinal Microbiota Affect Central Levels of Brain-Derived Neurotropic Factor and Behavior in Mice. Gastroenterology.

[84] Zhang H.X., Liu M., Zhong W.L., Zheng Y.P., Li Y.N., Guo L.P., Zhang Y., Ran Y., Zhao J., Zhou L. (2021). Leaky Gut Driven by Dysbiosis Augments Activation and Accumulation of Liver Macrophages RIP3 Signaling Pathway in Autoimmune Hepatitis. Front. Immunol..

[85] Feng Z., Long W.M., Hao B.H., Ding D., Ma X.Q., Zhao L.P., Pang X. (2017). A human stool-derived strain caused systemic inflammation in specific-pathogen-free mice. Gut Pathogens.

[86] Natividad J.M., Lamas B., Pham H.P., Michel M.L., Rainteau D., Bridonneau C., da Costa G., Van Hylckama Vlieg J., Sovran B., Chamignon C. (2018). Bilophila wadsworthia aggravates high fat diet induced metabolic dysfunctions in mice. Nat. Commun..

[87] Caso J.R., MacDowell K.S., Gonzalez-Pinto A., Garcia S., de Diego-Adelino J., Carceller-Sindreu M., Sarramea F., Caballero-Villarraso J., Gracia-García P., De la Cámara C. (2021). Gut microbiota, innate immune pathways, and inflammatory control mechanisms in patients with major depressive disorder. Transl. Psychiatry.

[88] Olson C.A., Iniguez A.J., Yang G.E., Fang P., Pronovost G.N., Jameson K.G., Rendon T.K., Paramo J., Barlow J.T., Ismagilov R.F. (2021). Alterations in the gut microbiota contribute to cognitive impairment induced by the ketogenic diet and hypoxia. Cell Host Microbe.

[89] Reichenberg A., Yirmiya R., Schuld A., Kraus T., Haack M., Morag A., Pollmächer T. (2001). Cytokine-associated emotional and cognitive disturbances in humans. Arch. Gen. Psychiatry.

[90] Sominsky L., Fuller E.A., Bondarenko E., Ong L.K., Averell L., Nalivaiko E., Dunkley P.R., Dickson P.W., Hodgson D.M. (2013). Functional programming of the autonomic nervous system by early life immune exposure: Implications for anxiety. PLoS ONE.

[91] Duivis H.E., Vogelzangs N., Kupper N., de Jonge P., Penninx B.W. (2013). Differential association of somatic and cognitive symptoms of depression and anxiety with inflammation: Findings from the Netherlands Study of Depression and Anxiety (NESDA). Psychoneuroendocrinology.

[92] Chung J.M., Kim H.K., Chung K. (2004). Segmental spinal nerve ligation model of neuropathic pain. Methods Mol. Med..

[93] Ji G., Yakhnitsa V., Kiritoshi T., Presto P., Neugebauer V. (2018). Fear extinction learning ability predicts neuropathic pain behaviors and amygdala activity in male rats. Mol. Pain..

[94] Shen C.L., Wang R., Ji G., Elmassry M.M., Zabet-Moghaddam M., Vellers H., Hamood A.N., Gong X., Mirzaei P., Sang S. (2022). Dietary supplementation of gingerols- and shogaols-enriched ginger root extract attenuate pain-associated behaviors while modulating gut microbiota and metabolites in rats with spinal nerve ligation. J. Nutr. Biochem..

[95] Ji G.C., Fu Y., Ruppert K.A., Neugebauer V. (2007). Pain-related anxiety-like behavior requires CRF1 receptors in the amygdala. Mol. Pain.

[96] Shen C.L., Santos J.M., Elmassry M.M., Chen F., Ji G.C., Presto P., Kiritoshi T., Liu X., Neugebauer V. (2025). Crosstalk Among Gut Microbiota, Fecal Metabolites, and Amygdala Neuropathology Genes After Ginger Polyphenol Administration in Female Rats with Neuropathic Pain: Evidence for Microbiota-Gut-Brain Connection. Nutrients.

[97] Rao X., Huang X., Zhou Z., Lin X. (2013). An improvement of the 2^(-delta delta CT) method for quantitative real-time polymerase chain reaction data analysis. Biostat. Bioinforma Biomath..

