# Supplementary material for: Gingerol-Enriched Ginger Extract Effects on Anxiety-like Behavior in a Neuropathic Pain Model via Colonic Microbiome-Neuroimmune Modulation
Source: Molecules. 2026 Jan 1;31(1):166. doi: 10.3390/molecules31010166 (PMC12787805; doi:10.3390/molecules31010166)
Supplement: Supplementary file 1 [file molecules-31-00166-s001.zip › molecules-3997800-supplementary.pdf]

Table S1. List of primers for mRNA

Gene	Forward	Reverse
NRF2	5'-CTC TCT GGA GAC GGC CAT GAC-3'	5'-CTG GGC TGG GGA CAG TGG TAG T-3'
CX3CR1	5'-TTC CTG CAG AAG TCC CCG TC-3'	AAG GCC ACG ATG TCA CCC AA-3'
MEK1	5'-TCC GCG GTT AAC GGG ACC AG-3'	GAA CAC CAC TCC ACC ATT GCC-3'
LDHA	5'-GAG CCA CTG TCG CCG ATC TC-3'	CCA ACA GCA CCA ACC CCA AC-3'
HIF1α	5'-TTT CTC TGC GCG TGA GGA CA-3'	TCG ACG TTC GGA ACT CAT CCT-3'
GPM6A	5'-TTG GAC AAG CGG ACT GCA CG-3'	GCC ACA GAA CAG GGC AAC G-3'
GLUT1	5'-ACT GTG GTG TCG CTG TTC GT-3'	AGG ACC GGG GCC TAC TTC AA-3'
SLC1A3	5'-CGG AGA AGA GCC CAG GAT GG-3'	TAC GGT CGG AGG GCA AAT CC-3'
SLC1A2	5'-CTC GTC GCC ACT GTC TCC AG-3'	GGT TTC GGT GCT TTG GCT CC-3'
GRM5	5'-ACT CAG CCT AGT TTA TCC AGC TGT T-3'	GCC AAA TGA TGC CCT GCG TT-3'
GRIN2C	5'-ACC TGG CAC TCC TGC AAC TT-3'	AGC ATG TAG AAG ACC CCC GC-3'
GRIN2B	5'-ATG GCC CTC AGC CTC ATC AC-3'	CAC GGA TTG GCG CTC CTC TA-3'
GluRA	5'-AAC TGA ACG AGC AGG GGC TT-3'	CCA GTC CCA GCC CTC CAA TC-3'
CHRNA7	5'-TGG TCC TAT GGA GGG TGG TCA-3'	ACG GCG CAT GGT TAC TGT GT-3'
5-HT3A	5'-CGT GCT GAT AGC CCA GGG AG-3'	TCC ACG TTG AGG ATG GCA TAC A-3'
5-HT2A	5'-GGT CAT CAT GGC AGT GTC CC-3'	GCT TGC TAG GCA AAG GCC AC-3'
MAO-A	5'-GCC TAT GTG GGA CCA ACC CA-3'	ATG CCA AGG GGT TCC ACA CA
SIRT1	5'-GCT ACC GAG ACA ACC TCC TGT T-3'	ATT GTT CGA GGA TCG GTG CCA
IDO1	5'-TCT TGC CGT TCC CTA CTG CG-3'	GCC TTT ATC GCA GTC CCC AC
KMO	5'-TGG CAT CGT CGG ACA CTG AA-3'	CAA GGC CTG CCG TCC TCT AT
HAAO3	5'-AGC CTG TTT GGG GAC AGC TA-3'	GCT CCC ACA TGT ACG AGA ACC
B-actin	5'-ACA ACC TTC TTG CAG CTC CTC C-3'	5'-TGA CCC ATA CCC ACC ATC ACA-3'
